# Immobilization of the Tannase From *Aspergillus fumigatus* CAS21: Screening the Best Derivative for the Treatment of Tannery Effluent Using a Packed Bed Reactor

**DOI:** 10.3389/fbioe.2021.754061

**Published:** 2021-11-03

**Authors:** Rayza Morganna Farias Cavalcanti, Chadia Chahud Maestrello, Luis Henrique Souza Guimarães

**Affiliations:** ^1^ Instituto de Química de Araraquara—UNESP, Araraquara, Brazil; ^2^ Departamento de Biologia, Faculdade de Filosofia, Ciências e Letras de Ribeirão Preto—USP, Ribeirão Preto, Brazil

**Keywords:** tannin acyl hydrolase, alginate beads, tannery wastewater, enzymatic reactor, effluent treatment

## Abstract

Enzyme immobilization is an important alternative to stabilize enzyme properties favoring the efficiency of derivatives (enzyme + support/matrix) for different purposes. According to this, the current study aimed to immobilize the *Aspergillus fumigatus* CAS21 tannase and the use of the derivatives in the treatment of the effluent produced by the tannery industry. The tannase was immobilized on sodium alginate, DEAE-Sephadex, amberlite, and glass pearls as supports. Calcium alginate was the most adequate support for tannase immobilization with 100% yield and 94.3% for both efficiency and activity. The best tannase activity for the calcium alginate derivative was obtained at 50°C–60°C and pH 5.0. Thermal and pH stabilities evaluated for 24 h at 30°C–60°C and pH 4–7, respectively, were improved if compared to the stability of the free enzyme. Considering the reuse of the calcium alginate derivative, 78% of the initial activity was preserved after 10 catalytic cycles, and after the 9-month storage at 4°C, the activity was maintained in 70%. This derivative was applied in a packed bed reactor (PBR) for the treatment of tannin-rich effluents from the tannery industry. The reduction of the tannin content was effective reaching degradation of 74–78% after 48 h of PBR operation. The concentration of total phenolic compounds was also reduced, and the color and clarity of the effluent improved. In conclusion, the calcium alginate derivative is an attractive alternative as biocatalyst for large-scale treatment of the effluents from the tannery industry.

## Introduction

The manufacture of leather is one of the most widespread processes worldwide due to the fact that it is an input in the industries of footwear, clothing, accessories, furniture, and automotive items (Dixit et al., 2015). The leather is obtained from the tanning process of animal skin, usually coming from bovine, equine, and caprine sources, supplied by beef cattle and slaughterhouses (Saxena et al., 2017). In general, the process involves the stages of preparation, tanning, and finishing (Dixit et al., 2015). The preparatory phase is cleaning the leather and eliminating parts that will not be used, such as removing the animal hair (Dixit et al., 2015; Mosca Angelucci et al., 2017). Most tanneries use salt to restrict microbial attack and lime to remove hair (Kanagaraj et al., 2015). In the tanning process, vegetable/synthetic tannins or mineral agents (aluminum, chromium, and zirconium) are applied in order to avoid skin putrefaction (Laurenti et al., 2016). These compounds provide greater durability of leather and stabilization of the collagen present in animal skin, avoiding chemical, thermal, and microbiological degradation (Onem et al., 2015). The finishing stage includes rinsing the tanned leather, drying, cutting, and concluding the final aspects to improve its commercial value (Mosca Angelucci et al., 2017).

The tannery industry is classified as one of the most polluting industries, generating large amounts of environmentally harmful liquids through all stages of the industrial process (Saxena et al., 2017). One of the main constituents of the effluents released by the tannery industries is the synthetic or vegetable tannins. This type of effluent impacts negatively on the soil, leading to deficiencies in the nutrients required by plants and promoting the inhibition of the microbial growth, thus modifying the local biota (Zhang et al., 2014; Kumar et al., 2018). Specifically, tannins slow down the process of humus formation and inhibit the enzymatic activity of soil microorganisms (Sharma, 2019). Contamination of the natural reservoirs of water by tannin-rich effluents makes the water unsuitable for supply. The aquatic life is also affected, since the high concentration of tannins is toxic to a variety of microorganisms and animals (Murugan and Al-Sohaibani, 2010; Zhang et al., 2014; Dixit et al., 2015).

The methods used to treat wastewater from the tannery industry are generally biological, chemical, and/or a combination of these methods. However, the presence of tannins, sulfides, and high salinity inhibits microbial growth and hinders the efficiency of biological treatment (Mosca Angelucci et al., 2017). Tannin inactivates extracellular enzymes, forms cross-links with compounds present in the membranes of microbial cells, and inhibits the growth of fungi and bacteria (Govindarajan et al., 2016; Balakrishnan et al., 2018). Efficient, clean, and environmentally sustainable methodologies attract worldwide attention to overcome the limitations of the effluent treatment processes by biological and chemical methods (Dixit et al., 2015). A viable alternative is the use of microbial enzymes. These biocatalysts can act efficiently and selectively to degrade target pollutants. In this context, the tannase supplementation can assist biological treatments, minimizing environmental impacts caused by the excessive presence of tannins and improving the biological degradation of wastewater (Murugan and Al-Sohaibani, 2010).

Tannase (tannin acil hydrolase TAH; EC 3.1.1.20) hydrolyzes esters and depsidic bonds of complex and hydrolyzable tannins, such as tannic acid, releasing glucose, and gallic acid (Govindarajan et al., 2016; Dhiman et al., 2018). This enzyme shows a potential of application in the food and beverage sector, in the chemical and pharmaceutical industries, in animal feed, and in the treatment of effluents with high tannin concentration (Kumar et al., 2018).

One of the most important challenges in the enzyme technology for industrial purposes is the stabilization of the enzymatic activity, which can be obtained through enzyme immobilization procedures (physical adsorption, ionic and covalent bonding, and encapsulation or entrapment). Immobilized enzymes have improved properties regarding the reaction temperature and pH, thermal and pH stability, greater catalytic performance, and storage stability (Larosa et al., 2018). Enzymatic immobilization also provides advantages such as the reuse of the derivative by repeated catalytic cycles, easy handling and product separation, and improved enzymatic stability. Reuse promotes savings in enzyme, time, investment, and labor (Andrade et al., 2020). These improved properties allow the use of derivatives in a packed bed reactor (PBR) (Wu et al., 2016; Ong and Annuar, 2018).

The immobilization of fungal tannases using different supports and matrices has been reported as for the enzyme produced by *Aspergillus niger* (Flores-Maltos et al., 2011), *Aspergillus aculeatus* (El-Tanash et al., 2011), and *Apergillus awamori* (Kumar et al., 2015), with significant improvement of the enzymatic properties. The fungus *Aspergillus fumigatus* CAS21 was reported as an interesting source of tannase with potential application in propyl gallate synthesis and effluent treatment (Cavalcanti et al., 2018). Hence, this enzyme is an attractive molecule to be stabilized through an immobilization process aiming at the obtainment of a better catalyst.

Considering the importance of the enzyme technology for the white biotechnology, i.e., the application of biological system enzymes instead of chemical catalysts at the industrial scale reducing the environmental impact (Heux et al., 2015; Barcelos et al., 2018), this manuscript describes, for the first time, the obtaining of a catalyst through the immobilization of the *A. fumigatus* CAS 21 tannase to be used in the treatment of tannin-rich effluents using the PBR system.

## Materials and Methods

### Microorganism and Culture Conditions

The endophytic fungus *Aspergillus fumigatus* CAS21 was isolated from the bark of the cashew tree (*Anacardium occidentale* L.) (Cavalcanti et al., 2017) and stored in the Laboratory of Microbiology of the Faculdade de Filosofia, Ciências e Letras de Ribeirão Preto, University of São Paulo, Brazil. The fungus was maintained on potato dextrose agar (PDA) slants at 37°C for 96 h and then stored at 4°C. The liquid cultures were obtained by means of the inoculation of 1 ml of spore suspension (10^5^ spores/ml) of the *A. fumigatus* CAS21 in 25 ml of mineral medium containing 0.1% (w/v) peptone and 2% (w/v) tannic acid as additional carbon source (Cavalcanti et al., 2017). The culture medium was previously autoclaved at 120°C and 1.5 atm for 20 min, and the tannic acid was sterilized by microfiltration using a syringe filter (0.22 μm) before the addition to the medium.

The cultures were kept at 37°C for 24 h at 120 rpm. After cultivation, the medium was harvested using a vacuum pump and Whatman filter paper No. 1. The cell-free filtrate containing tannase was dialyzed overnight for 24 h at 4°C against distilled water and applied on a Millipore membrane with a 50-kDa cutoff, submitted to centrifugation at 2,800 × *g* at 4°C for 15 min. The enzyme retained in the permeate was used for enzymatic immobilization.

### Enzymatic Immobilization

The tannase was immobilized using the methods of encapsulation, covalent bonding, and adsorption, in the supports sodium alginate, amberlite IR 140, glass pearls, and DEAE-Sephadex A25. The immobilization process was accompanied by the determination of the enzymatic activity and by the quantification of supernatant proteins.

### Encapsulation in Cross-Linked Sodium Alginate With CaCl_2_ and MnCl_2_

An aqueous solution of sodium alginate was prepared in distilled water at 4% (m/v) concentration and shaken for 4 h at 28°C. Then, the solution was mixed with tannase to yield a final concentration of 3% (m/v) alginate and gently shaken for 15 min at 4°C. The suspension was dripped, with the aid of a syringe (10 ml), into the CaCl_2_ solution (0.1 mol L^−1^). The formed beads (Ca-alginate) were gently stirred in CaCl_2_ solution for 20 min at 4°C, then collected and washed extensively with sodium acetate buffer (100 mmol L^−1^ pH 5.0). The same procedure was performed replacing CaCl_2_ by MnCl_2_ (0.1 mol L^−1^) (Mn alginate). The beads were stored in sodium acetate buffer at 4°C, and then the enzymatic activity was quantified.

### Covalent Bonds

#### Alginate Activated With Glutaraldehyde

Alginate beads activated with glutaraldehyde (GA) were prepared according to the methodology described by Pal and Khanum (2011), with modification. The beads obtained in the presence of CaCl_2_, as described above, were activated in 9% GA (v/v in 10 mmol L^−1^ pH 5.0 sodium acetate buffer), in the proportion 1:1 (m/v), for 2 h. The activated beads were washed with sodium acetate buffer and added with 3 ml of enzymatic solution. The mixture was kept in gentle agitation for 120 min at 4°C. The derivatives were collected, washed with sodium acetate buffer to remove the unbound enzyme, and stored at 4°C in the same buffer.

#### Amberlite Activated With GA

The amberlite IR 140 activated with GA was prepared according to the methodology described by Kumari and Kauastha (2011), with modification. One gram of amberlite IR 140 balls was balanced in 4 ml of sodium acetate buffer (10 mmol L^−1^ pH 4.0) and subjected to agitation at room temperature for 2 h. Then, the sodium acetate buffer was removed and the beads were activated with 2 ml of 2.5% (v/v) GA for 2 h under agitation. The GA solution was removed by vacuum filtration; the beads were washed extensively with sodium acetate buffer (10 mmol L^−1^ pH 4.0), and then 10 ml of enzymatic solution was added. The suspension was kept in gentle agitation for 16 h, at 4°C. The derivative was recovered by filtration and washed twice with sodium acetate buffer (10 mmol L^−1^ pH 4.0) to remove the unbound proteins.

#### Glass Bead Activated With GA

Glass beads (5 g) were activated in 4 ml of GA 9% (v/v) (GA), previously prepared in sodium acetate buffer (10 mmol L^−1^ pH 5.0) (Chang et al., 2006). The suspension was kept under agitation for 2 h at room temperature. Then, the glass beads were filtered, washed extensively with sodium acetate buffer (10 mmol L^−1^ pH 5.0), and added with 10 ml of enzyme solution. The suspension was kept under agitation for 16 h at 4°C. The glass beads were recovered and washed with sodium acetate buffer to remove the unbound proteins.

#### Adsorption in DEAE-Sephadex Resin A25

One gram of DEAE-Sephadex A25 resin was balanced in 20 ml of sodium acetate buffer (10 mmol L^−1^ pH 5.0) and shaken at room temperature for 2 h. Afterward, the buffer was removed and 10 ml of enzymatic solution was added. The suspension was kept in agitation for 16 h, at 4°C. Afterward, the derivative was vacuum filtered and washed three times with sodium acetate buffer (10 mmol L^−1^ pH 5.0) to remove the unbound proteins and then stored at 4°C in the same buffer.

#### Determination of the Tannase Activity and Protein Quantification

The tannase activity was determined using 0.2% (w/v) methyl gallate as substrate in 100 mmol L^−1^ sodium acetate buffer (pH 5.0) according to the methanolic rhodanine method (Sharma et al., 2000). For immobilized tannase, the reaction was composed of 2 ml of substrate and derivative (1 g of sodium alginate and glass beads; 0.5 g of amberlite derivative; 0.1 g of DEAE-Sephadex A25 derivative). After 5 min of reaction, aliquots of 500 μl were transferred to reaction tubes and 300 μl of 0.667% (w/v) methanolic rhodanine was added. After 5 min, 200 μl of 0.5 N potassium hydroxide was added and the mixture was maintained at 30°C. Finally, the mixture was diluted with 4 ml of distilled water and incubated at 30°C for 10 min. The absorbance was recorded at 520 nm using a spectrophotometer (UV Mini-1240, Shimadzu, Kyoto, Japan). One unit (U) of enzymatic activity was defined as the amount of enzyme necessary to produce 1 μmol of gallic acid per minute under the assay conditions.

The protein quantification was performed according to the Bradford method (Bradford, 1976) using bovine serum albumin (BSA) (Sigma-Aldrich, St. Louis, MO, USA) as standard.

#### Determination of Yield, Efficiency, and Recovered Activity

The immobilization yield (Y%) (Eq. 1) and efficiency (E%) (Eq. 2), as well as the recovered activity (R%) (Eq. 3), were calculated according to Sheldon and Pelt (2013):

$$Y\%=\frac{\mathrm{Immobilized}\;\mathrm{activity}}{\mathrm{Start}\;\mathrm{activity}}\times 100$$ (1)

$$E\%=\frac{\mathrm{Observed}\;\mathrm{activity}}{\mathrm{Immobilized}\;\mathrm{activity}}\times 100$$ (2)

$$R\%=\frac{\mathrm{Observed}\;\mathrm{acivity}\;}{\mathrm{Start}\;\mathrm{activity}}\times 100$$ (3)

The immobilized activity was determined by the difference between the total initial activity and the enzyme activity that remains in the solution after the immobilization process. The activity observed is the enzymatic activity of the derivative after immobilization.

### Characterization of the Immobilized Enzyme

#### Effect of Temperature on Enzyme Activity and Thermal Stability

The effect of temperature on the enzymatic activity of both the free tannase and the derivative was determined by conducting the enzymatic reactions at temperatures from 30°C to 80°C. The highest activity was considered as 100% and was used as reference. Thermal stability was determined by incubating the free tannase and the derivative at 30°C, 40°C, 50°C, and 60°C for 24 h. Every 2 h, aliquots were collected, kept in an ice bath, and then screened for tannic activity. The enzymatic activity at time 0 was considered as 100%.

#### Effect of pH on Enzyme Activity and pH Stability

The effect of pH on the enzymatic activity of the free tannase and the derivative was determined using 100 mmol L^−1^ citric acid buffer (pH 3.0 and 4.0), 100 mmol L^−1^ sodium acetate buffer (pH 5.0 and 6.0), 100 mmol L^−1^ Tris–HCl buffer (7.0), and 100 mmol L^−1^ glycine (8.0). The highest activity was considered as 100% and used as reference. In order to assess the pH stability, the free enzyme and the derivative were incubated in the abovementioned buffer solutions in the pH range of 4.0–7.0 at 4°C for 24 h and then enzyme activity was measured at a standard assay condition every 2 h.

#### Kinetic Parameters of the Ca-alginate Derivative

The kinetic constant of the Ca-alginate derivative was determined at different concentrations of tannic acid (0.001–5.0 mM) as substrate. The reactions were conducted at pH 5.0 and 50°C. The *K*_*m*_ and *V*_*max*_ values were calculated using the software SIGRAF (Leone et al., 1992).

#### Derivative Reuse and Storage Stability

The reuse of the Ca-alginate derivative was investigated by measuring its activity after repeated catalytic cycles under optimized enzymatic assay conditions (temperature and pH). After each cycle, the derivative was washed with sodium acetate buffer (100 mmol L^−1^ pH 5.0) to remove any residual substrate and products. A new substrate solution was employed after each cycle, and the enzymatic activity was determined according to *Encapsulation in Cross-Linked Sodium Alginate With CaCl*_*2*_ *and MnCl*_*2*_. The enzymatic activity of the first cycle was defined as 100%.

The storage stability of the Ca-alginate derivative was checked by measuring the residual activity after 9 months (274 days) stocked in sodium acetate buffer (100 mmol L^−1^ pH 5.0) at 4°C.

#### Enzymatic Treatment of Tannery Effluent in a Packed Bad Reactor

The leather effluent samples were supplied by Cooperativa Arteza, Paraíba, Brazil. The effluents come from the manual tanning process (MTP) of goat leather treated with angico burk (*Anadenanthera colubrina* Vell.) in tanks for 15 days.

The synthetic effluent was prepared simulating the effluent obtained from the tanning process performed with synthetic tannin, as described by Panizza and Cerisola (2004), with modifications. The simplified synthetic effluent (SSE) was composed of 6 g L^−1^ tannic acid (Synth), 7 g L^−1^ NaCl, and 8 g L^−1^ Na_2_SO_4_ in distilled water.

#### Operation in a Packed Bed Reactor With Ca-alginate Derivative

**FIGURE 1:**
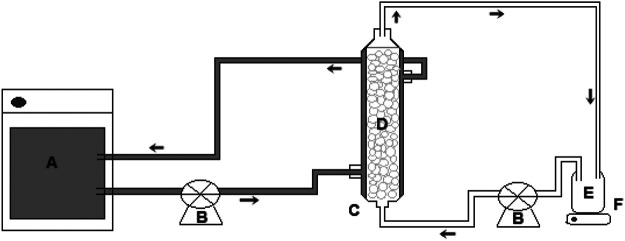
Packed bed reactor system: **(A)** thermostatic bath, **(B)** flow control valve, **(C)** PBR column, **(D)** immobilized biocatalyst, **(E)** effluent, **(F)** magnetic stirrer.

Figure 1 shows a schematic representation of the experimental system used for the treatment of tannery effluent in PBR. The experimental apparatus consisted of a glass reactor (internal diameter of 2.5 cm and length of 15 cm), equipped with an external jacket for recirculation of water at a controlled temperature of 37°C. The process was carried out in a discontinuous regime with recirculation. The feed solution was pumped through the column in an ascending flow mode and with a flow rate of 5 ml min^−1^ controlled by a peristaltic pump. The feed solution was kept under magnetic stirring for homogenization at room temperature (28°C). The enzymatic derivative was retained in the reactor using a polyethylene screen deposited at the upper end of the PBR.

The reactor was filled with 35.65 g of Ca-alginate derivative, and 150 ml of the effluent was deposited in the feed/recirculation tank. The treatment was conducted for 48 h at 37°C. The properties of the Ca-alginate derivative are presented in Table 1. Samples of effluents were collected (2 ml) every 2 h after the start of the process, and the total tannin concentration was determined. The Ca-alginate beads without the addition of enzyme were used as control in the effluents to verify the spontaneous hydrolysis of the tannins, and the process was conducted following the same procedure mentioned above. Treated and untreated effluents were characterized through determination of the tannin content, total phenols, color, clarity, and pH.

**TABLE 1:** Properties of the Ca-alginate derivative used in PBR for the treatment of the effluents MTP and SSE.

Properties	MTP	SSE
Wet mass (g)	35.6	35.7
Enzymatic activity (total U)	275.6 ± 5.3	234.2 ± 17.0
Density (g ml^−1^)	1.0 ± 0	1.0 ± 0
Bead diameter (cm)	0.4 ± 0	0.4 ± 0

MTP: manual tanning process; SSE: simplified synthetic effluent.

#### Operational Parameters of Process in Packed Bed Reactor

The spatial time or residence time (Ʈ) of the packed bed reactor was calculated according to Eq. 4.

$$Τ=\frac{V}{V_{0}}$$ (4)

where Ʈ = space time (min), V is the working volume of the reactor (ml), and V_o_ is the volumetric flow rate (ml. min^−1^).

The working volume of the reactor was calculated after subtracting the volume of the reactor covered with the enzyme immobilized (V_catalyst_). V_catalyst_ was calculated as described in Eq. 5.

$$V_{\mathrm{catalyst}}=\frac{w}{\rho }$$ (5)

The spatial velocity (S) corresponds to the number of reactor volumes that were fed under specified conditions and that can be treated in a unit of time (h^−1^) (Eq. 6).

$$S=\frac{1}{\tau }$$ (6)

The void fraction (Ɛ) of the bed or porosity was defined by the ratio between the volume of the bed that is not occupied with the catalyst and the total volume of the bed.

#### Quantification of Tannin and Phenolic Compounds

The tannin content was determined by the protein precipitation method (Hagerman and Butler, 1978). Determination of the total phenolic compounds was performed using the Folin–Ciocalteu reagent, according to Rao et al. (2018).

#### Color, Clarity, and pH Determination

The color and clarity of the treated and untreated effluents were determined by measuring the absorbance at 420 nm (Abs 420) and transmittance at 660 nm (T% 660), respectively, in a spectrophotometer. Distilled water was used as control (Rai and De, 2009; Lima et al., 2018a). The pH was measured using a pH meter, Fisher Scientific Accumet^®^ AB15 Basic model.

#### Statistical Analysis

Microsoft Office Excel 2007 (Microsoft) and OriginPro 8 (OriginLab Corporation, Trial version) programs were used for data analysis and graphical representations. The assays were performed in triplicates and analyzed based on mean ± standard error of the samples.

## Results

### Immobilization of the *A. fumigatus* CAS21 Tannase

Table 2 presents the results of the immobilization of tannase produced by *A. fumigatus* CAS21 using encapsulation, covalent binding, and adsorption methods. Among all the supports studied, Ca-alginate was the best matrix to immobilize *A. fumigatus* CAS21 tannase with 100% yield, 94.3% efficiency, and 94.3% recovered activity. The second best derivative was the Mn-alginate with 100% yield, efficiency of 81.3, and 81.3% recovered activity. These results indicate that the enzyme was efficiently encapsulated in the alginate beads and no activity was detected in the supernatant. Considering the covalent binding, the best yield was observed for the GA-alginate derivative (100%), but with reduced tannase activity as also observed for GA-amberlite and GA-glass beads. The adsorption using DEAE-Sephadex as support allowed good values of yield (92.8%) and 49.5% efficiency, but reduced activity.

**TABLE 2:** Immobilization of *A. fumigatus* CAS21 tannase using different methods and supports.

Methods	Immobilization yield (%)	Efficiency (%)	Recovered activity (%)
Encapsulation			
Ca-alginate	100 ± 0	94.3 ± 3.8	94.3 ± 3.8^A^
Mn-alginate	100 ± 0	81.3 ± 61	81.3 ± 6.1^B^
Covalent bond			
GA-alginate	100 ± 0	34.6 ± 2.8	34.6 ± 2.8^C^
GA-amberlite	81.6 ± 1.3	18.0 ± 1.8	14.7 ± 1.3^D^
GA-glass pearls	78.0 ± 2.6	35.6 ± 0.8	27.8 ± 1.6^C^
Adsorption			
DEAE-sephadex	92.1 ± 0.8	49.5 ± 4.5	45.6 ± 4.2^E^

GA glutaraldehyde. Values with the same letter in the column do not differ under Tukey´s test at 5% probability (*p* ≤ 0.05).

### Effects of the Temperature and pH on Tannase Activity of the Ca-alginate Derivative

**FIGURE 2:**
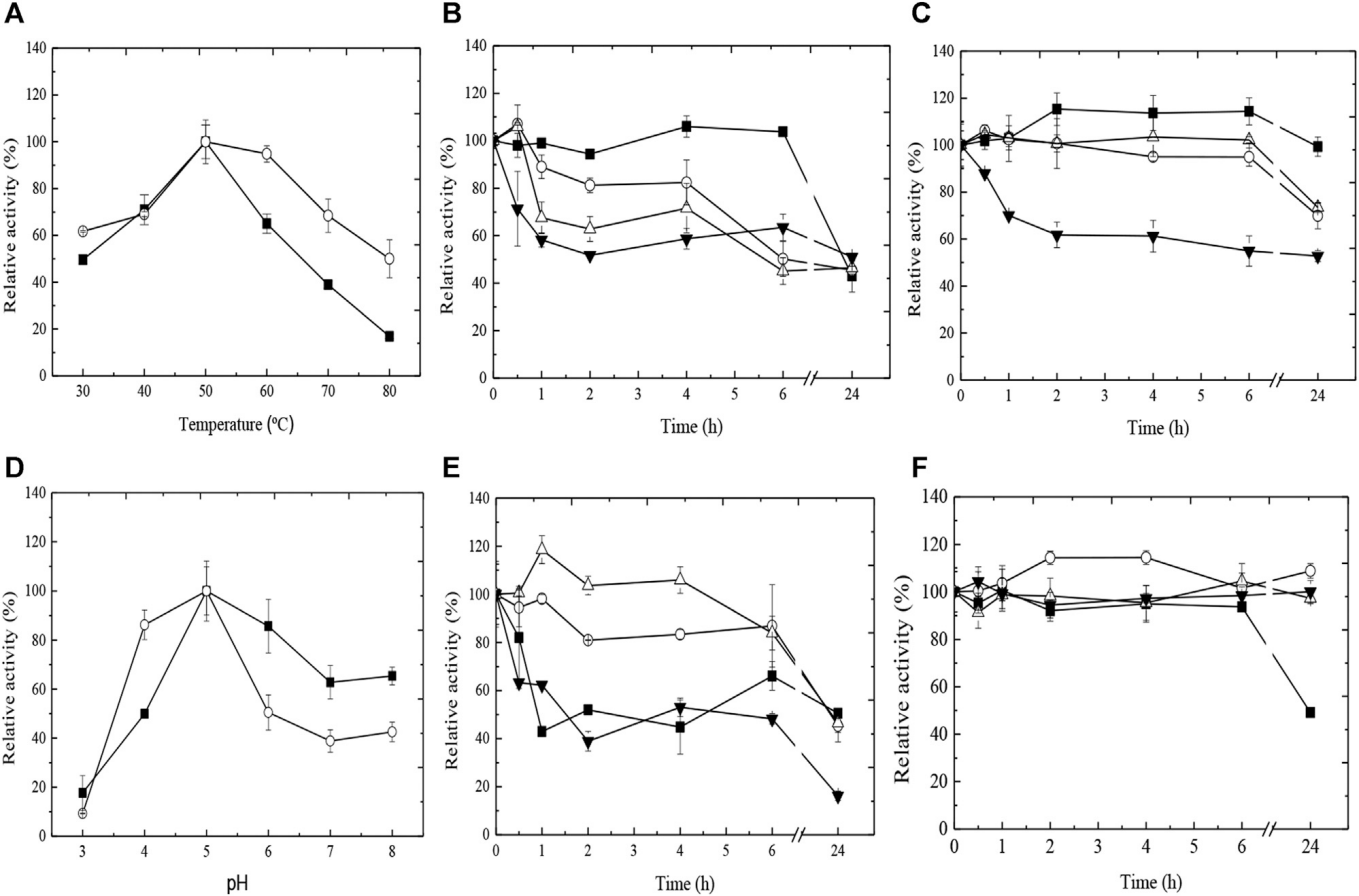
Effect of temperature **(A)** and pH **(D)** on the enzymatic activity of free (■) and immobilized (○) tannases from *A. fumigatus* CAS21. Thermal **(B, C)** and pH **(E, F)** stabilities of free **(B–E)** and immobilized **(C–F)** tannases from *A. fumigatus* CAS21. Symbols: for **(B, C)**, 30°C (■), 40°C (○), 50°C (∆), and 60°C (▼) for 24 h; for **(E, F)**, pH 4.0 (■), 5.0 (○), 6.0 (∆), and 7.0 (▼) for 24 h.

Considering that the best yield, efficiency, and tannase activity were obtained with the Ca-alginate derivative, the influence of temperature and pH on enzymatic activity was analyzed (Figure 2). The free tannase exhibited maximal activity at 50°C, and the increase in temperature caused a gradual reduction in enzymatic activity, with a decrease of 83% at 80°C. On the other hand, the best temperature for the enzymatic activity of the Ca-alginate derivative was achieved at 50°C–60°C. At 80°C, 50% of initial activity was maintained (Figure 2A). In addition, the Ca-alginate derivative exhibited better thermal stability at high temperatures compared to the free enzyme (Figures 2B,C). The half-life (t_50_) for the free enzyme at 50°C was 6 h, while the Ca-alginate derivative was fully stable from 30°C to 50°C for 6 h. At 40°C and 50°C, 70% of the derivative activity was observed with 24 h of incubation, and t_50_ of 24 h at 60°C.

Considering the influence of pH on the enzymatic activity, both free enzyme and Ca-alginate derivative presented the best activity at pH 5.0 (Figure 2D). However, the pH stability profiles for both enzymatic forms were different (Figures 2E,F). Around 80% of the free tannase activity was maintained at pH 5.0 and 6.0 for 6 h, with reduction to 50% with 24 h of incubation. At pH 4.0 and 7.0, t_50_ was 1–1.5 h. The Ca-alginate derivative showed superior pH stability for all periods of incubation analyzed, preserving its initial activity when incubated for 24 h at pH 5.0, 6.0, and 7.0. At pH 4.0, t_50_ was 24 h.

### Kinetic Parameters

The Michaelis constant (*K*_*m*_) can vary depending on the enzyme and substrates. In this study, the *K*_*m*_ calculated for the Ca-alginate derivative showed an apparent value of 0.236 mM and a maximum velocity (*V*_*max*_) of 183.40 U mg^−1^.

### Reuse and Storage Stability

**FIGURE 3:**
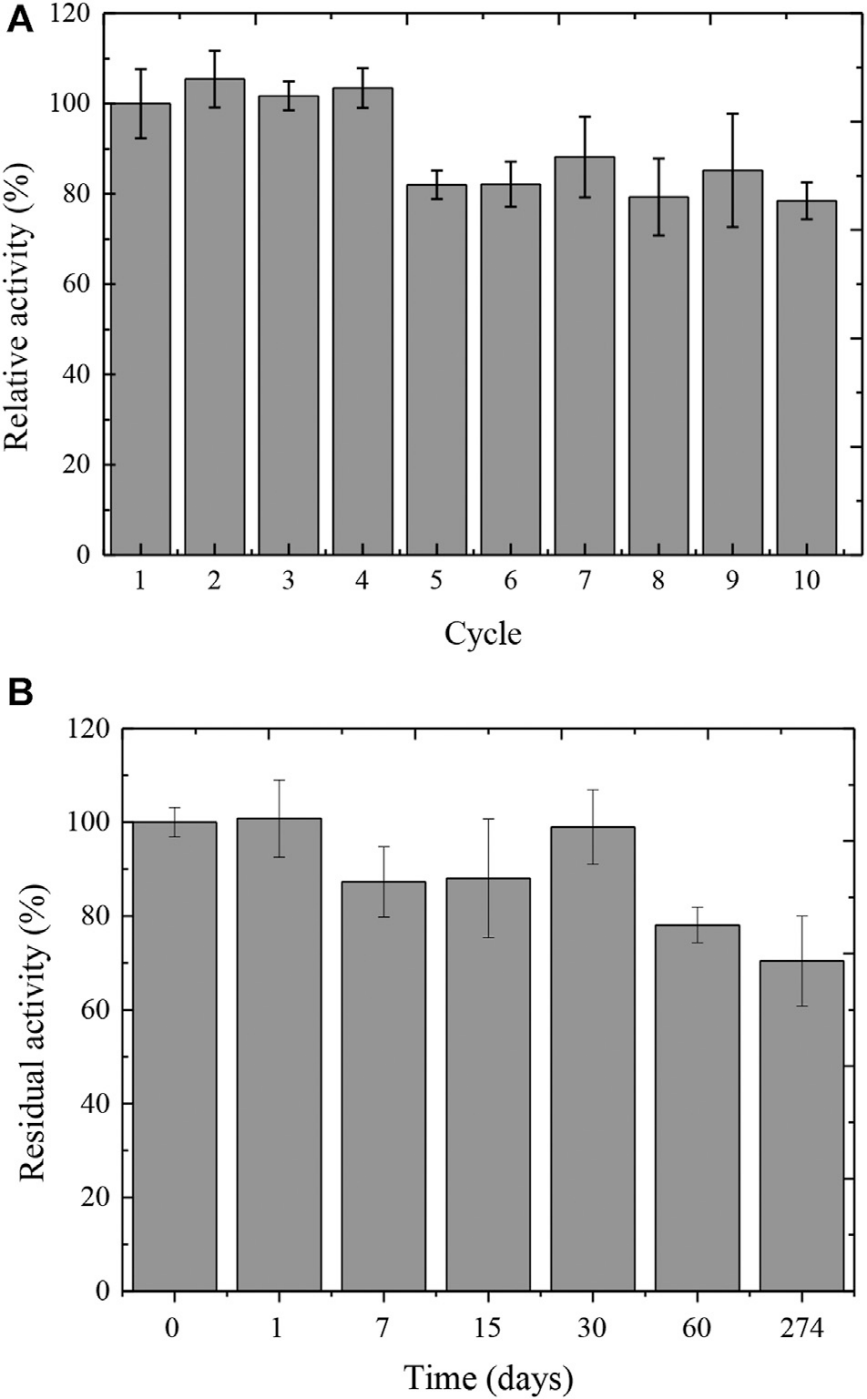
Reuse **(A)** and storage stability **(B)** of the Ca-alginate derivative containing *A. fumigatus* CAS21 tannase.

The Ca-alginate derivative was analyzed regarding the reuse cycles with reactions performed at 50°C and pH 5.0. The derivative retained 100% of the initial tannase activity after the fourth cycle of reuse (Figure 3A). At the fifth cycle, the activity was maintained around 78% and remained up to 10 cycles of continuous application.

The storage stability of the Ca-alginate derivative was analyzed for 9 months (274 days) at 4°C, and a high enzymatic activity of the derivative was observed for 30 days, with 99% retention of its initial value (Figure 3B). Interestingly, 70% of derivative activity was obtained after 9 months of storage.

### Enzymatic Treatment of Tanning Industry Effluent in a Packed Bed Reactor

The process parameters for the treatment of tannery effluent in the PBR at 37°C for 48 h, with a recycling system and a flow rate at 5 ml min^−1^, are described in Table 3. The spatial time in the reactor was 7.7 min for MTP and 7.6 min for SSE. Therefore, 7.8 and 7.9 reactor volumes were fed into the reactor per hour in the treatment of effluents MTP and SSE, respectively. The void fraction of bed (expressed as porosity) depends on the size and shape of the particles. For each process, the size and shape of the alginate beads were the same and, consequently, the calculated porosity as well (0.5). The particles had diameters of 0.4 cm (Table 1). After 24 h of operation, the enzymatic activities of the Ca-alginate derivative were maintained at 96.8% and 84.6% considering the MTP and SSE treatments.

**TABLE 3:** Operational parameters of PBR process for the treatment of MTP and SSE effluents from the tanning industry using Ca-alginate derivative containing *A. fumigatus* CAS21 tannase.

Parameters	MTP	SSE
Ʈ (min)	7.7	7.6
S (h^−1^)	7.8	7.9
Ɛ	0.5	0.5
RA (%)	96.8 ± 1.5	84.6 ± 8.9

Ʈ: spatial time; S: space velocity; Ɛ: porosity of the bed; AR: residual activity; MTP: manual tanning process; SSE: simplified synthetic effluent.

**FIGURE 4:**
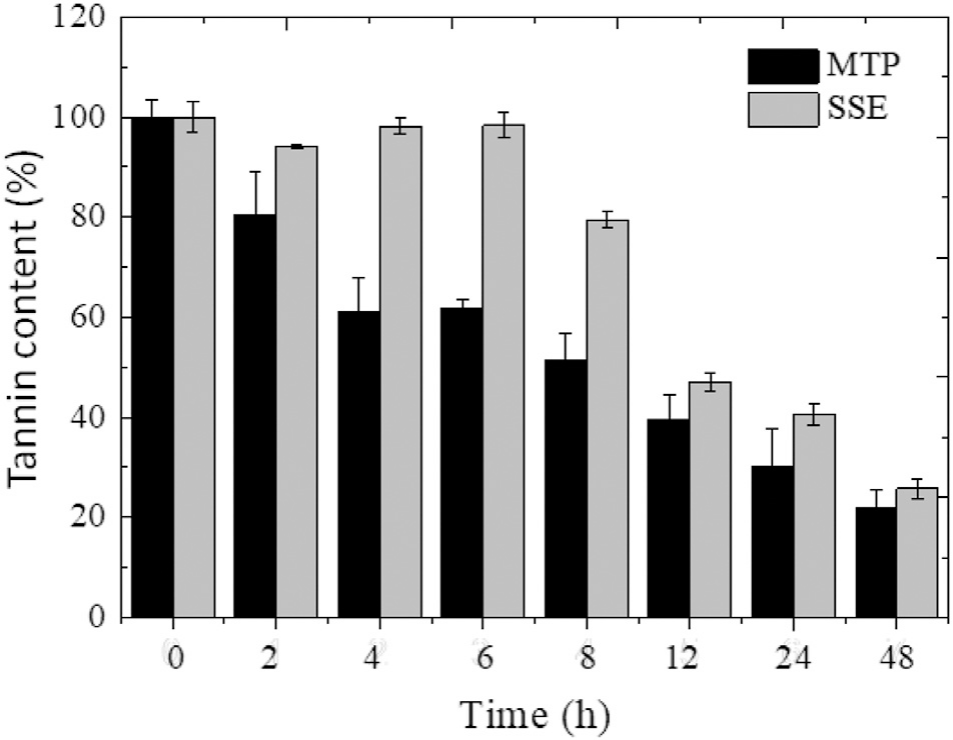
Time-course of the MTP and SSE effluents using Ca-alginate derivative containing *A. fumigatus* CAS21 tannase in the PBR for 48 h at 37°C with a flow rate of 5 ml min^−1^.

Figure 4 depicts the profile of the reduction of the tannin contents of the MTP and SSE for 48 h of enzymatic treatment using the Ca-alginate derivative. After 2 h of operation of the PBR, around 20% of the tannin content of the MTP was hydrolyzed, reaching 50% reduction after 8 h. In contrast, no significant reduction in tannin content was observed for SSE after 6 h of treatment. With treatment periods from 8 h, the tannin content of the SSE was gradually reduced. The enzymatic treatment of the effluents using a compacted bed bioreactor resulted in the effective removal of 78.1% and 74.3% of tannin content from the MTP and SSE, respectively, after 48 h of operation. An increase in the period of treatment up to 120 h did not promote a significant reduction in the concentration of tannins in the effluents if compared to the treatment performed for 48 h (data not presented), indicating stabilization of the process.

The physical–chemical characteristics of the untreated and treated effluents are presented in Table 4. The treatment using the Ca-alginate derivative reduced 70.3% and 48.3% of the phenolic compounds present in the MTP and SSE effluents, respectively. The color of the effluents was reduced about 48.7% for MTP and 62.4% for SSE, and the clarity increased 6.7-fold and 1.33-fold for MTP and SSE, respectively.

**TABLE 4:** Physical–chemical characteristics of MTP and SSE effluents before and after treatment using Ca-alginate derivative containing *A. fumigatus* CAS21 tannase in the PBR.

Parameters	Untreated MTP	Treated MTP	Untreated SSE	Treated SSE
Tannin (%)	100 ± 5.5^A^	21.9 ± 3.4^B^	100 ± 3.0^a^	25.72 ± 1.9^b^
Phenolic (mg of GAE/mL)	0.6 ± 0.0^A^	0.2 ± 0.0^B^	0.6 ± 0.0^a^	0.3 ± 0.0^b^
Color (A_420nm_)	20.1 ± 0.2^A^	10.3 ± 0.1^B^	1.3 ± 0.1^a^	0.5 ± 0.0^b^
Clarity (T_660%_)	7.7 ± 0.4^A^	50.9 ± 0.4^B^	61.5 ± 0^a^	80.2 ± 10.7^b^
pH	7.2	7.8	3.8	4.0

Values with the same letter in line do not differ under Tukey’s test at 5% probability (*p* ≤ 0.05). MTP: manual tanning process; SSE: simplified synthetic effluent.

## Discussion

Tannases are enzymes with biotechnological potential to be applied in different areas such as beverage and food, feed manufacturing, chemical and pharmaceutical industries, and treatment of rich tannin effluents (Jana et al., 2014). Microorganisms as filamentous fungi are important sources of tannases. The *A. fumigatus* CAS 21 tannase was reported as an interesting biocatalyst with potential to be applied in propyl galate synthesis and in the treatment of leather effluent (Cavalcanti et al., 2018). This fact highlights this enzyme as an attractive candidate to be immobilized, aiming at the improvement of its properties.

One of the most important attributes considered for an effective enzyme use in different enzymatic processes is stability, which can be obtained using the immobilization technology, improving their properties. The type of support and the method used for the immobilization can influence the enzyme activity and its biochemical properties (Yao et al., 2014). Here, the immobilization of *A. fumigatus* CAS21 tannase using different methodologies was described. The best results considering efficiency, yield, and recovered activity were obtained for Ca-alginate derivatives, which were higher than those described for most of the tannases immobilized using the same procedure. For example, the *A. niger* tannase immobilized in alginate retained only 37.6% of activity after optimization of the immobilization conditions (Li et al., 2004). Lima et al. (2018a) obtained 70% efficiency and 75% recovered activity for the Ca-alginate derivative containing commercial *A. ficuum* tannase. The immobilization of a tannase from a metagenomic library using calcium alginate beads allowed an immobilization yield of 62% (Yao et al., 2014). However, according to Andrade et al. (2020), the efficiency of immobilization of the *Penicillium rolfsii* tannase using alginate beads was 99.5%, similar to the result described here. It is important to highlight that the results obtained for *A. fumigatus* CAS21 tannase immobilization in alginate beads were more expressive than those observed for the other supports used. The tannase immobilization using superparamagnetic ferroferric oxide nanoparticles allowed only 14.71% of recovered activity (Wu et al., 2016), while the use of amino-functionalized magnetic Fe_3_O_4−_ chitosan nanoparticles allowed 32.28% of activity retention (Li et al., 2018). Immobilization yields of 77.6% and 63.1% were reported for the use of Dowex 50W and chitin as supports, respectively (Kumar et al., 2015).

The highest activity observed for the Ca-alginate derivative containing *A. fumigatus* CAS21 tannase can be explained by the unaltered structure of the enzyme, since there is no chemical connection with the support. This method is considered as simple and low-cost, and the immobilization is often carried out under mild conditions of temperature and pH (Larosa et al., 2018). Additionally, the alginate polymer presents important physicochemical properties for industrial application, such as biocompatibility, biodegradability, and non-toxicity (Brus et al., 2017).

Covalent immobilization of the *A. fumigatus* CAS21 tannase using calcium alginate, amberlite, and glass beads activated by GA showed the lower efficiencies and activities (less than 34.6% recovered activity). The recovered activity observed for the activated glass bead derivative was statistically similar to that found for the activated calcium alginate derivative in spite of the minor yield value. The hydroxyl groups of the glass bead surface allow the reaction with the GA. The aldehyde groups react with the amino groups of the amino acid residues on the enzyme surface (Wu et al., 2016). The covalent bond methodology promotes structural rigidity and stability of the enzyme. However, the occurrence of the conformational changes can cause loss or reduction of enzymatic activity (Wu et al., 2016), as observed in this work. The linkage of the enzyme to the GA-activated support may have occurred in the region close to the active site, which makes access to the substrate difficult. Aharwar and Parihar (2021) reported a reduction in the activity of the *Talaromyces verruculosus* tannase immobilized in alginate activated by GA, which was explained due to the blockade of the active site or by the leaching of the enzyme. In addition, the covalent bond may also modify the enzyme structure, decreasing its catalytic activity (Wu et al., 2016). The covalent immobilization of the *A. aculeatus* tannase allowed a better immobilization yield than the immobilization using calcium alginate, differing from our results (El-Tanash et al., 2011).

Considering the best results obtained using the Ca-alginate derivative, the influence of temperature and pH on tannase activity was analyzed. The temperature of activity observed for both free and Ca-alginate derivatives was the same differing from the observation of other authors. For example, Lima et al. (2018b) described the best temperatures of 40°C for the immobilized tannase in nanoparticles of diatomaceous Earth and 30°C for the free form. The best temperature of reaction for the *A. awamori* tannase immobilized in amberlite was 55°C, while for the free form it was achieved at 30°C (Kumar et al., 2015). Stability profiles obtained with different temperatures indicate that Ca-alginate immobilization increased the thermal resistance of *A. fumigatus* CAS21 tannase. The same was observed by Lima et al. (2018a) for the *A. ficuum* tannase immobilized in Ca-alginate. Immobilized enzymes have the potential to operate at temperatures around 10°C higher than those reported for the free form and thermally stable enzymes are promising for industrial application (Basso and Serban, 2019).

The best pH of activity (5.0) was the same for both free and Ca-alginate derivatives. Similar to our results, the optimum pH of activity for both the immobilized tannase in diatomaceous nanoparticles and the free enzyme from *A. ficuum* was the same (6.0) (Lima et al., 2018b). Sometimes, alterations in the pH of activity between the free and immobilized enzymes can be observed, as reported for the *A. awamori* tannase, with the best pH of activity changing from 4.0 for the free enzyme to 5.5 for the amberlite derivative (Kumar et al., 2015). The pH stability results for the Ca-alginate derivative containing *A. fumigatus* CAS21 tannase are consistent with those obtained for other immobilized tannases. Andrade et al. (2020) reported that the tannase of *P. rolfsii* CCMB 714 immobilized in alginate was more stable than the free enzyme in a wide range of pH (2.0–5.0), retaining more than 50% of the residual activity for 16 h. Aharwar and Parihar (2021) observed an increase in the stability of the *T. verruculosus* tannase at pH 8.0 and 9.0 for 12 h after immobilization in alginate beads. Li et al. (2004) reported that the *A. niger* tannase immobilized in alginate was stable at a wide pH range (2.0–7.0) compared to the free enzyme (3.0–5.5). The acidic pH of activity and stability observed for the Ca-alginate derivative containing *A. fumigatus* CAS21 tannase suggests that it may be of great value in tea and juice processing.

The kinetic parameters can also be modified considering both the free and immobilized enzymes. This fact was observed for the enzyme from *A. fumigatus* CAS21. The *K*_*m*_ and *V*_*max*_ values obtained for hydrolysis of the tannic acid using the Ca-alginate derivative were lower than those obtained for the free *A. fumigatus* CAS21 tannase (*K*_*m*_ of 6.38 and *V*_*max*_ of 360.2 U mg^−1^) as reported by Cavalcanti et al. (2018). The decrease in *K*_*m*_ after immobilization may indicate increased binding affinity to the substrate compared to the free enzyme. According to Kumar et al. (2015), this occurs due to a change in the microenvironment around the active site under immobilized conditions. Similar results were observed for *A. tubingensis* CICC 2651 tannase immobilized in ferroferric oxide nanoparticles (Wu et al., 2016) and for *A. awamori* tannase immobilized in amberlite (Kumar et al., 2015). In addition, encapsulated enzymes tend to have lower *V*_*max*_ as a result of the resistance that the matrix imposes for substrate diffusion into the beads (Li et al., 2004). Kumar et al. (2015) and Li et al. (2004) reported *V*_*max*_ lower than free tannase for enzymes immobilized on amberlite and alginate, respectively.

The reuse and storage stability of the Ca-alginate derivative was investigated aiming a better understanding of its biotechnological potential. The successful application of enzymes in industrial processes requires high operational stability (Jana et al., 2015). Compared to other supports, the immobilization in alginate beads allows a fast recovery by filtration or screening, generating cost reduction of the global process. The operational stability reported in this study was higher than that reported for other immobilized tannases. The *A. ficuum* tannase immobilized using alginate beads retained only 35% of activity after nine catalytic cycles (Lima et al., 2018a), while the *P. rolfsii* CCMB 714 tannase retained 50% of activity over six cycles (Andrade et al., 2020). The *T. verruculosus* tannase maintained 42.9% of the initial activity after 10 cycles of reuse (Aharwar and Parihar, 2021). On the other hand, the derivative containing the T410 tannase, from a metagenomic library, had its activity reduced only 20% after 26 cycles (Yao et al., 2014). Additionally, the results herein reported were better than those described for other types of support as magnetized nanoparticles (Wu et al., 2016) and magnetic chitosan nanoparticle (Li et al., 2018).

The immobilization of *A. fumigatus* CAS21 tannase using Ca-alginate beads improved the storage stability. Enzymes in solutions are most unstable for long periods of storage due to reactions with oxygen and also with the storage solvent (Jana et al., 2015), while immobilization by encapsulation avoids direct contact of the enzyme with the external environment. Similarly, tannase immobilized on magnetized nanoparticles maintained 96% of the initial activity after 30 days of storage at 4°C (Wu et al., 2016). Jana et al. (2015) described that the chitin–alginate derivative preserved 83% of the tannase activity after 90 days of storage at 4°C. Lima et al. (2018a) reported that the alginate derivative retained 50% of tannase activity after 105 days at 4°C.

Physical characteristics such as tolerance to a wide range of pH and temperature and reuse for different consecutive cycles make the Ca-alginate derivative containing *A. fumigatus* CAS21 tannase a promising system for the treatment of wastewater with tannins.

### Enzymatic Treatment of the Tannin-Rich Effluent From the Leather Industry

The efficiency of the biological treatment of wastewater produced by the leather industry can be improved by adding a primary phase of enzymatic degradation of tannins, followed by the biological treatment for the decomposition of other constituents (Balakrishnan et al., 2018). In this context, the use of tannases to hydrolyze tannins provides gallic acid and glucose as hydrolysis products and non-toxic and biodegradable substances, more susceptible to subsequent treatment (Murugan and Al-Sohaibani, 2010).

Aiming at the application of the Ca-alginate derivative for the treatment of effluent with high tannin content produced by the leather industry using PBR, some operational parameters of the process were determined as spatial time and velocity, bead porosity, and residual activity. The spatial time indicates the time required to process a feed volume and is related to the reactor feed flow rate, while the spatial velocity defines how many reactor volumes of feed can be treated per hour.

The particle size of the Ca-alginate derivative containing the *A. fumigatus* CAS21 tannase was higher than that of the minimal value (0.05 mm) considered to avoid the problem with the compaction of the bead and close to that reported by Oliveira et al. (2018) for spherical particles. During the conduction of the PBR process, no clogging of the system was observed, which can be attributed to the particle size of the beads. According to Poppe et al. (2015), to avoid clogging and non-standard pressure drop, the supports used to immobilize enzymes must have diameters greater than 0.05 mm.

The enzymatic treatment of the effluents (both MTP and SSE) through the PBR process with the Ca-alginate derivative allowed significant reduction (74%–78%) in the tannin content after 48 h. The potential of some tannases to reduce the tannin content of the effluents has been described. In this context, the free and spray-dried *A. fumigatus* CAS21 tannase hydrolyzed tannins and phenolic compounds present in the tannery effluent (Cavalcanti et al., 2018; Cavalcanti et al., 2020). Sharma and Saxena (2012) described that the *A. niger* and *P. variable* tannases showed degradation of 45% and 36% of the tannins present in the tannery effluent after 48 h, respectively. The *A. ficuum* tannase reduced 57% of the tannin content present in the effluents of the tanning process after 24 h of treatment (Balakrishnan et al., 2018). However, this is the first description of the use of the PBR process with the Ca-alginate derivative containing tannase for the leather effluent treatment.

The hydrolysis of tannins by the action of tannase is essential to reducing the degree of toxicity of the effluents from the tannery industry. Hansen et al. (2020), studying the cytotoxicity of the chemicals present in the leather industry effluents on fibroblasts of V79-4 cells, found 60.1% acute toxicity provoked by natural tannins and 50%–60% toxicity in chronic tests. The synthetic tannins showed 100% toxicity in both exposure tests. Additionally, the authors stated that tannery compounds (natural and synthetic tannins) are responsible for the greatest pollution load of wastewater. The use of derivatives containing tannase in enzymatic reactors is an interesting alternative for the treatment of effluents with high phenolic compounds.

According to Cavalcanti et al. (2018) and Aracri et al. (2019), the free tannases produced by the fungi *A. fumigatus* CAS21 and *A. ochraceus* promoted a reduction of about 30% and 64%, respectively, of phenolic compounds of the leather effluents. The use of the Ca-alginate derivative containing *A. fumigatus* CAS21 tannase allowed better reduction of the phenolic compounds of the MTP and SSE compared to that mentioned for the free enzyme.

Other parameters as color and clarity should also be considered for the analysis of the treated effluents. The wastewater produced by the tanning industry is characterized by its cloudy appearance and dark coloring, which is green or dark brown when chrome is used in tanning (Durai and Rajasimmam, 2011; Saxena et al., 2017). The dark coloring prevents the penetration of sunlight, thus reducing photosynthesis and inhibiting the biota growth (Carpenter et al., 2013). The intense color of the water is considered as a problem for the treatment of these samples. A high degree of clarity of an effluent indicates a low turbidity index and, consequently, a lower degree indicates the concentration of the solids present. In this context, the hydrolysis of the tannins by the Ca-alginate derivative reduced the concentration of insoluble solids in the effluents, providing increased clarity. Similar results were reported by Murugan and Al-Sohaibani (2010). These authors noted that the enzymatic treatment of the effluent from the mango industry using *A. candidus* MTTC 9628 tannase promoted considerable reduction of the tannin content as well as color by 55%. Color and clarity are parameters that indicate the degree of the wastewater quality.

The use of enzymatic hydrolysis as a pretreatment of effluents can improve their biological treatment. According to Vashi et al. (2018), the biological treatments are characterized as long-time and ineffective processes for the significant removal of tannins. In the activated sludge model, about 24% of the tannins were removed after 72 h of processing (Balakrishnan et al., 2018). In spite of the high reduction (97%) of the tannin content of the pulp and paper industry effluents, 10 days were necessary for the obtainment of this result using the aerobic granular sludge technology (Vashi et al., 2018).

## Conclusion

The immobilization by entrapment in alginate beads was shown to be a promising technique for the immobilization of *A. fumigatus* CAS21 tannase. The thermal and pH stabilities of the tannase were considerably enhanced for the Ca- alginate derivative, improving the catalytic potential of the tannase and highlighting this derivative as an efficient biocatalyst for large-scale biotechnological applications. The Ca-alginate derivative containing tannase is an appropriate biocatalyst to be used in the packed bed reactor (PBR) for the treatment of tannery effluents. In addition, the Ca-alginate derivative can be reused for successive operational cycles in PBR. Our findings suggest that the treatment of leather industry effluents in the PBR using Ca-alginate derivative is a very attractive alternative for the reduction of tannin and phenolic compound contents, and for the clarification of wastewater. After the enzymatic treatment, the effluent showed improved and adequate characteristics to increase the efficiency of subsequent treatment steps, such as biological treatment.

## Data Availability

The raw data supporting the conclusion of this article will be made available by the authors, without undue reservation.
